# Association of Body Composition With Survival and Treatment Efficacy in Castration-Resistant Prostate Cancer

**DOI:** 10.3389/fonc.2020.00558

**Published:** 2020-04-17

**Authors:** Sahyun Pak, Myeong Seong Kim, Eun Young Park, Sung Han Kim, Kang Hyun Lee, Jae Young Joung

**Affiliations:** ^1^Department of Urology, Center for Urologic Cancer, Research Institute and Hospital, National Cancer Center, Goyang-si, South Korea; ^2^Department of Radiology, National Cancer Center, Research Institute and Hospital, Goyang-si, South Korea; ^3^Biostatistics Collaboration Team, Research Core Center, Research Institute and Hospital, National Cancer Center, Goyang-si, South Korea

**Keywords:** castration-resistant prostate cancer, body composition, survival, treatment efficacy, disease progression

## Abstract

**Objectives:** The association of body composition with survival and the efficacy of first-line treatment was investigated in patients with castration-resistant prostate cancer (CRPC).

**Methods:** The records of CRPC patients treated with docetaxel or androgen receptor signaling inhibitors (ARSi) between 2005 and 2018 were reviewed. Skeletal muscle index (SMI), visceral fat index, and subcutaneous fat index were evaluated using pretreatment computed tomography images.

**Results:** Of 230 eligible patients, 144 received docetaxel, and 86 received ARSi as the first-line treatment for CRPC. The SMI^hi^ (based on median values) group had higher prostate-specific antigen (PSA) progression-free survival (median 13.5 vs. 8.3 months, *p* = 0.030), radiologic progression-free survival (14.9 vs. 9.1 months, *p* < 0.001), and overall survival (24.1 vs. 16.9 months, *p* = 0.015) than the SMI^lo^ group. In docetaxel-treated patients, the SMI^hi^ group had higher PSA progression-free survival (13.5 vs. 5.9 months, *p* = 0.016) and radiologic progression-free survival (14.6 vs. 6.7 months, *p* < 0.001) than the SMI^lo^ group. However, PSA progression-free survival and radiologic progression-free survival were comparable between the two groups in ARSi-treated patients. SMI was independently associated with the risk of radiologic progression in patients treated with docetaxel but not in those treated with ARSi.

**Conclusions:** High skeletal muscle mass may be associated with reduced risk of disease progression and mortality in patients with CRPC. However, the significance of these relationships is limited in patients treated with docetaxel. These results suggest that assessing skeletal muscle mass may be worthwhile when selecting treatments for CRPC; however, further prospective validation and large-scale studies are needed.

## Introduction

The treatment of castration-resistant prostate cancer (CRPC) remains a major challenge despite the availability of several therapeutic options including docetaxel-based chemotherapy and androgen signaling inhibitors (ARSi) (1). The optimal treatment strategy remains to be fully determined, underscoring the importance of predicting the response to treatment in patients with CRPC (2).

The clinical implications of individual body composition in patients with cancer have been investigated extensively, and a relationship between cancer patient survival and muscle mass or fat mass has been identified (3, 4). In addition, body composition is associated with disease progression and treatment response in cancer patients (5–7).

Studies evaluating CRPC patients who underwent chemotherapy reported that muscle mass and subcutaneous fat mass are associated with overall survival (8–10). However, the survival outcomes according to body composition in CRPC patients who received first-line ARSi were not clearly determined. Moreover, the relationship between body composition and disease progression or treatment efficacy remains to be investigated. Here, we investigated the association between body composition and disease progression, mortality, and efficacy of first-line treatment in patients with CRPC.

## Methods

### Study Population and Design

The records of 314 men diagnosed with CRPC who underwent first-line therapy at the National Cancer Center Hospital between January 2005 and June 2018 were reviewed. Patients who did not undergo adequate abdominopelvic computed tomography (CT) scanning before starting first-line treatment (*n* = 75), those treated for <2 months (*n* = 5), and those followed-up for <6 months (*n* = 4) were excluded. The study protocol was approved by the Institutional Review Board of the National Cancer Center (IRB No. NCC2018-0123). The study design followed all relevant principles of the Declaration of Helsinki.

Demographic characteristics, clinical characteristics, laboratory findings, radiologic findings, pathologic features, and survival outcomes were evaluated retrospectively. The following parameters were examined: age, treatment type, pretreatment prostate-specific antigen (PSA) level, metastatic disease status, presence of pain, secondary treatment, diabetes, performance status, biopsy tumor grade, alkaline phosphatase, lactate dehydrogenase, hemoglobin levels, and body composition indices. All patients underwent androgen deprivation therapy before castration-resistant disease progression. Before starting first-line treatments for CRPC, all patients underwent height and weight measurements, blood tests, and abdominopelvic CT. Patients were not randomly assigned to docetaxel or ARSi arms. Treatment selection was determined by the treating physician and patients. The chemotherapy treatment protocol comprised docetaxel (75 mg/m^2^ once every 3 weeks) and prednisolone (5 mg twice daily). Enzalutamide was administered orally at a dose of 160 mg per day, and abiraterone was administered orally at a dose of 1,000 mg per day along with prednisolone (5 mg twice daily).

According to the follow-up protocol, the serum concentration of PSA was measured monthly, and abdominopelvic CT and bone scanning were performed every 3–6 months. Docetaxel or ARSi treatments were continued until the emergence of evidence of PSA progression, radiologic progression, or severe treatment-related adverse events.

The primary endpoint was radiologic progression-free survival. Secondary endpoints were PSA progression-free survival and overall survival. PSA progression and radiologic progression were determined by serum PSA tests and imaging studies in accordance with Prostate Cancer Working Group 2 (PCWG2) criteria (11).

### Body Composition Assessments

Muscle mass and subcutaneous and visceral fat mass were evaluated using pretreatment CT images. Skeletal muscle index (SMI; cm^2^/m^2^), subcutaneous fat index (SFI; cm^2^/m^2^), and visceral fat index (VFI; cm^2^/m^2^) were calculated based on the height and cross-sectional area of the skeletal muscle, subcutaneous fat, and visceral fat at the 3rd lumber vertebra (12). The cross-sectional CT images of each patient were extracted from a picture archiving and communication system (PACS) and analyzed using AWS 2 (GE Healthcare, Chicago, IL, USA) by two readers. SMI, SFI, and VFI were dichotomized at median values.

### Statistical Analysis

Clinicopathological data were presented as means or medians for continuous variables and as frequencies for categorical variables. Between-group differences in categorical variables were assessed with the Chi-square test, and differences in continuous variables were assessed with the Student's *t*-test or the Wilcoxon rank sum test. PSA progression-free survival, radiologic progression-free survival, and overall survival were estimated using the Kaplan–Meier method, and survival curves between groups were compared using the log-rank test. The association between variables and outcomes was assessed by multivariable analysis using a Cox proportional hazards model. Covariates, chosen based on previous studies, were adjusted to group variables. Optimal cut-off levels for SMI, VFI, and SFI were defined using the median values of each index, and the Contal and O'Quigley method (Table S1). Because the results of two methods showed similar cut-off points, each index was dichotomized into high and low indices based on median values.

Propensity score matching was used to ensure a similar distribution of covariates between groups. The effect of group on prognosis was then evaluated in the dichotomized subset based on median values.

All statistical tests were two-tailed, and statistical significance was defined as p < 0.05. All statistical analyses were performed using SAS 9.4 (SAS Institute Inc., Cary, NC, USA) and R project software 3.5.1.

## Results

### Patient Characteristics

Of 230 eligible patients who were diagnosed with CRPC, 144 received docetaxel chemotherapy and 86 received ARSi (enzalutamide: 61; abiraterone: 25) as first-line treatment for CRPC (Table 1). The median follow-up was 21.3 months. Baseline characteristics were different between docetaxel-treated and ARSi-treated patients except tumor grade, primary treatment, and metastatic status, which were similar between the two groups. The median time on therapy was shorter for patients treated with docetaxel than for patients treated with ARSi (4.6 vs. 11.0 months, respectively). Median overall survival was 17.7 months in patients treated with docetaxel and 25.0 months in those treated with ARSi. Secondary treatments were administered to 61.8 and 61.6% of docetaxel-treated and ARSi-treated patients, respectively.

**Table 1:** Baseline characteristics of patients.

		**First-line treatment**		
	**Overall (*****n*** **=** **230)**	**Docetaxel (*****n*** **=** **144)**	**ARSi (*****n*** **=** **86)**	***P*****-value**
Age (years, mean)	68.3	66.3	71.8	<0.001
Year of treatment				<0.001
<2015	107 (46.5)	104 (72.2)	3 (3.5)	
≥ 2015	123 (53.5)	40 (27.8)	83 (96.5)	
Treatment duration (months, median)	6.0	4.6	11.0	<0.001
Diabetes	46 (20.0)	22 (15.3)	24 (27.9)	0.027
ECOG performance status				0.046
0	203 (88.6)	122 (84.7)	81 (95.3)	
≥1	26 (11.4)	22 (15.3)	4 (4.7)	
Gleason scores				0.174
6	3 (1.3)	2 (1.4)	1 (1.2)	
3+4	18 (7.8)	13 (9.0)	5 (5.8)	
4+3	36 (15.7)	17 (11.8)	19 (22.1)	
8	54 (23.5)	35 (24.3)	19 (22.1)	
9–10	105 (45.7)	65 (45.1)	40 (46.5)	
Unknown	14 (6.1)	12 (8.3)	2 (2.3)	
Primary treatment				0.109
None	182 (79.1)	113 (78.5)	69 (80.2)	
Radical prostatectomy	30 (13.0)	16 (11.1)	14 (16.3)	
Radiotherapy	18 (7.8)	15 (10.4)	3 (3.5)	
Time to castration-resistant (months, median)	18.3	20.7	14.8	0.041
Prostate-specific antigen (ng/ml, median)	56.9	69.3	47.6	0.015
Hemoglobin (g/dl, median)	11.8	11.7	12.0	0.138
Alkaline phosphatase (U/L, median)	246.1	254.2	234.0	0.638
Lactate dehydrogenase (U/L, median)	216.2	209.3	238.0	0.365
Body mass index (kg/m^2^, mean)	23.6	23.1	24.4	0.005
Skeletal muscle index (cm^2^/m^2^, median)	49.9	49.6	50.6	0.489
Visceral fat index (cm^2^/m^2^, median)	59.4	59.2	60.4	0.845
Subcutaneous fat index (cm^2^/m^2^, median)	48.2	46.7	52.0	0.025
Bone metastasis				0.657
None	34 (14.8)	21 (14.6)	13 (15.1)	
≤ 5 sites	53 (23.0)	36 (25.0)	17 (19.8)	
>5 sites	143 (62.2)	87 (60.4)	56 (65.1)	
Solid organ metastasis	28 (12.2)	20 (13.9)	8 (9.3)	0.405
Lymph node metastasis	122 (53.0)	70 (48.6)	52 (60.5)	0.081
Presence of pain	94 (40.9)	59 (41.0)	35 (40.7)	0.967
Secondary treatments				
None	88 (38.3)	55 (38.2)	33 (38.4)	
Docetaxel	16 (7.0)	0	16 (18.6)	
Enzalutamide	56 (24.3)	48 (33.3)	8 (9.3)	
Abiraterone	23 (10.0)	8 (5.6)	15 (17.4)	
Cabazitaxel	30 (13.0)	30 (20.8)	0	
Other treatments	5 (2.1)	3 (2.1)	2 (2.3)	
Unknown	12 (5.2)	0	12 (14.0)	

*ARSi, androgen receptor signaling inhibitor; ECOG, Eastern Cooperative Oncology Group*.

### Survival Outcomes According to Body Composition

**Figure 1:**
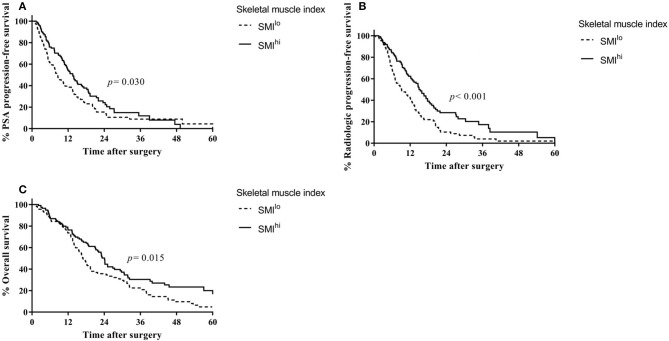
Outcomes based on skeletal muscle index in overall patients with castration-resistant prostate cancer from the time of diagnosis. **(A)** Prostate-specific antigen progression-free survival. **(B)** Radiologic progression-free survival. **(C)** Overall survival.

The median SMI, VFI, and SFI were 49.9, 59.4, and 48.2, respectively. Recurrence and survival outcomes based on SMI are shown in (Figure 1), and outcomes based on VFI and SFI are shown in (Figures S1, S2). The SMI^hi^ group had higher PSA progression-free survival (median, 13.5 vs. 8.3 months, *p* = 0.030; Figure 1A), radiologic progression-free survival (14.9 vs. 9.1 months, *p* < 0.001; Figure 1B), and overall survival (24.1 vs. 16.9 months, *p* = 0.015; Figure 1C) than the SMI^lo^ group, whereas the type of treatment was similar between the two groups (use of docetaxel: 60.0% vs. 65.2%, *p* = 0.414). In the multivariable analysis (Table S2), SMI was independently associated with risk of PSA progression [hazard ratio (HR) = 0.68; 95% confidence interval (CI), 0.50–0.93; *p* = 0.017], radiologic progression (HR = 0.54; 95% CI, 0.39–0.75; *p* = 0.001), and overall survival (HR = 0.72; 95% CI, 0.52–0.98; *p* = 0.037) regardless of BMI. Detailed results of the multivariable analyses for each measured outcome are shown in (Tables S2). To examine the robustness of the results, we performed sensitivity analyses. The results revealed that high SMI was associated with progression-free survival when patients were categorized into three tertiles based on SMI (Figure S3).

### Body Composition and Use of Docetaxel and Androgen Receptor Signaling Inhibitors

**Figure 2:**
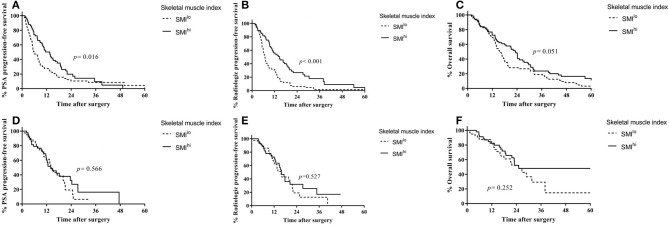
Outcomes according to first-line treatment based on skeletal muscle index in patients with castration-resistant prostate cancer. **(A)** Prostate-specific antigen progression-free survival in patients treated with docetaxel. **(B)** Radiologic progression-free survival in patients treated with docetaxel. **(C)** Overall survival in patients treated with docetaxel. **(D)** Prostate-specific antigen progression-free survival in patients treated with ARSi. **(E)** Radiologic progression-free survival in patients treated with ARSi. **(F)** Overall survival in patients treated with ARSi.

Overall, 144 patients were treated with docetaxel chemotherapy, with a median of six treatment cycles. In docetaxel-treated patients, PSA progression-free survival (13.5 vs. 5.9 months, *p* = 0.016; Figure 2A) and radiologic progression-free survival (14.6 vs. 6.7 months, *p* < 0.001; Figure 2B) were higher in the SMI^hi^ than in the SMI^lo^ group. Overall survival was also higher in patients with SMI^hi^ than in those with SMI^lo^ with near statistical significance (23.3 vs. 15.6, *p* = 0.051; Figure 2C). Time on therapy was shorter for the SMI^lo^ group than for the SMI^hi^ group, although the difference was not statistically significant (median, 4.1 vs. 5.2 months, respectively; *p* = 0.191). Overall, six patients discontinued chemotherapy due to toxicity (three patients in the SMI^lo^ group and three in the SMI^hi^ group). SMI^hi^ patients experienced fewer grade 3 and 4 adverse events than SMI^lo^ patients (44.6% vs. 63.5%, *p* = 0.044). The incidence of neutropenic fever was similar between patients with SMI^hi^ and SMI^lo^ (10.5% vs. 7.8%, *p* = 0.754).

In ARSi-treated patients, PSA progression-free survival (14.5 vs. 14.0 months, *p* = 0.566; Figure 2D), radiologic progression-free survival (15.9 vs. 15.6 months, *p* = 0.527; Figure 2E), and overall survival (25.1 vs. 25.0, *p* = 0.252; Figure 2F) were similar between the SMI^hi^ and SMI^lo^ groups.

After adjusting for covariates (Table S2), SMI^hi^ was associated independently with decreased risk of radiologic progression in patients treated with docetaxel (HR = 0.44; 95% CI, 0.30–0.65; *p* < 0.001) but not in those treated with ARSi (HR = 0.84; 95% CI, 0.46–1.54; *p* = 0.579). In ARSi-treated patients, SFI^hi^ was associated with decreased risk of radiologic progression (HR = 0.48; 95% CI, 0.24–0.94; *p* = 0.033).

To adjust clinical parameters prior to first-line treatment between patients in the docetaxel and ARSi groups, propensity score-based matching was performed (Table S3). HR values for radiologic progression derived from Cox models suggested that patients with SMI^lo^ are more likely to benefit from ARSi than docetaxel (Figure S4).

## Discussion

In this study, we found that skeletal muscle mass was associated with disease progression and survival after first-line therapy in patients with CRPC regardless of BMI. In addition, skeletal muscle mass was associated with the efficacy of docetaxel but not that of ARSi. These results suggest that assessment of muscle mass may be useful for first-line treatment selection in patients with CRPC.

Although several treatment options for CRPC have demonstrated a survival benefit and have been approved for clinical use, the optimal treatment strategy remains to be determined. Docetaxel and ARSi are the most widely used first-line treatments in patients with CRPC; however, randomized trial results have not been reported to date (13). Therefore, predictive markers of survival and treatment response in CRPC are critically needed.

The clinical implications of body habitus based on anthropometric measures have been intensively studied in various cancers (14–17). However, anthropometric measures such as BMI, waist circumference, or waist:hip ratio have limitations, and do not fully reflect muscle and fat mass (4). Recently, body composition has emerged as an alternative to traditional indicators. Body composition can be estimated easily and accurately using CT and software programs (12). Many studies report a significant association between body composition and survival of patients with cancer (3, 4). The relationship between body composition and prostate cancer was also investigated in those with localized and metastatic disease (8–10, 18, 19).

Subcutaneous adiposity has protective effects in cancer patients; potential explanations for this phenomenon include energy metabolism, adipose tissue signaling, and increased frequency of medical care (20). Previous studies report that subcutaneous fat mass is related to survival in CRPC patients treated with docetaxel (10) or ARSi (21). In this study, we found that a high subcutaneous fat mass was associated with better survival outcomes for ARSi-treated patients, which is consistent with the study by Antoun et al. (21).

In this study, we focused on the skeletal muscle mass. High skeletal muscle mass was significantly associated with a decreased risk of PSA progression, radiologic progression, and overall mortality in patients with CRPC. These relationships between SMI and survival outcomes remained significant in patients treated with docetaxel but not in those treated with ARSi. Cushen et al. examined body composition in CRPC patients treated with docetaxel and reported that low skeletal muscle mass was associated with an increased risk of not only chemotherapy toxicity, but also overall mortality (8). Ohtaka et al. also reported that sarcopenia is an independent factor that raises the risk of mortality (9). A recent study by Stangl-Kremser et al. demonstrated that sarcopenia is associated with tumor progression in CRPC patients (22). In addition, one study reported an association between muscle mass and survival in patients with non-metastatic prostate cancer (19). Although the underlying mechanisms remain unclear, skeletal muscle mass may be associated with insulin growth factor signaling, host immunity, systemic inflammation, and hormonal activity, which are related to the aggressiveness of prostate cancer (19). Moreover, accumulating evidence suggests that low muscle mass is associated with higher toxicity and poor tolerance to chemotherapy (7–10). Several studies reported that low muscle mass also contributes to decreased response to chemotherapy in patients with solid malignancies, including breast, colorectal, lung, and ovary cancer (7), which supports the present data.

ARSi drives changes in metabolic profiles and body composition. Previous studies demonstrate relationships between ARSi therapy and lipid metabolism and statin use (23–25). Both abiraterone and enzalutamide can induce increases in body fat mass and decreases in lean body mass (26, 27). However, the relationship between body composition and the response to ARSi in CRPC patients is largely unknown. One study reported that subcutaneous adiposity is associated with overall survival in metastatic CRPC patients treated with ARSi after chemotherapy (21). The present data indicate that the efficacy of first-line ARSi in CRPC patients is unrelated to skeletal muscle mass, unlike that of docetaxel. These results indicate that skeletal muscle mass may be helpful for first-line treatment selection and for predicting treatment responses in patients initially diagnosed with CRPC. Based on the results of this study, CRPC patients with low skeletal muscle mass should be considered for first-line ARSi therapy rather than docetaxel.

The present study has several limitations. First, the retrospective design may have caused inevitable inherent bias. The relatively short duration of follow-up and the lack of a standardized protocol for first-line and second-line treatment selection may be potential significant confounders and preclude analysis of overall survival. Second, the results of this study may not be applicable to other ethnic groups, such as Caucasians, because of ethnic and racial variations in body composition (4). Third, we did not analyze body composition changes during the treatment period. Individual changes in body composition may influence the efficacy of treatment (28). Although muscle or fat mass may be associated with prognosis or treatment response in those with CRPC, the mechanisms underlying this phenomenon and any causal relationships remain unclear; therefore, caution should be exercised when attempting to draw definitive conclusions from this study.

Despite these limitations, we believe that the present study provides valuable information to support the management of patients with CRPC. To the best of our knowledge, this study is the first to demonstrate differences in the treatment response to docetaxel or ARSi according to individual muscle mass and fat mass.

In conclusion, skeletal muscle mass was associated with PSA progression, radiologic progression, and mortality in patients initially diagnosed with CRPC. These relationships remained significant in patients treated with docetaxel but not in those treated with ARSi. These results suggest that treatment responses may differ according to muscle mass, and that assessing the SMI may be worthwhile when selecting first-line treatment and predicting responses to therapy. Large-scale prospective validation is warranted.

## Data Availability Statement

The datasets used and/or analyzed during the current study are available from the corresponding author upon reasonable request.

## Ethics Statement

This study protocol was approved by the Institutional Review Board of the National Cancer Center, Goyang, Korea. Written informed consent was not required.

## Author Contributions

SP designed the study, performed data analysis, and drafted the manuscript. MK performed data analysis. EP performed statistical analysis. SK and KL participated in data acquisition. JJ designed the study. All authors read and approved the final manuscript.

## Conflict of Interest

The authors declare that the research was conducted in the absence of any commercial or financial relationships that could be construed as a potential conflict of interest.

## Supplementary Material

Figure S1 Outcomes based on visceral fat index in overall patients with castration-resistant prostate cancer from the time of diagnosis. **(A)** Prostate-specific antigen progression-free survival. **(B)** Radiologic progression-free survival. **(C)** Overall survival.

Figure S2 Outcomes based on subcutaneous fat index in overall patients with castration-resistant prostate cancer from the time of diagnosis. **(A)** Prostate-specific antigen progression-free survival. **(B)** Radiologic progression-free survival. **(C)** Overall survival.

Figure S3 Outcomes based on skeletal muscle index tertiles in overall patients with castration-resistant prostate cancer from the time of diagnosis. **(A)** Prostate-specific antigen progression-free survival. **(B)** Radiologic progression-free survival. **(C)** Overall survival. 1st tertile: 26.73–46.92, 2nd tertile: 46.99–53.07, 3rd tertile: 53.09–77.24.

Figure S4 Treatment-specific hazards of radiologic progression.

Table S1 Cut-off points of body composition indices based on median values and the Contal and O'Quigley method.

Table S2 Multivariable analysis based on selected covariates.

Table S3 Characteristics of propensity score-matched patients.
