# Supplementary figures and images for: Association of Body Composition With Survival and Treatment Efficacy in Castration-Resistant Prostate Cancer
Source: Front Oncol. 2020 Apr 17;10:558. doi: 10.3389/fonc.2020.00558 (PMC7180747; doi:10.3389/fonc.2020.00558)

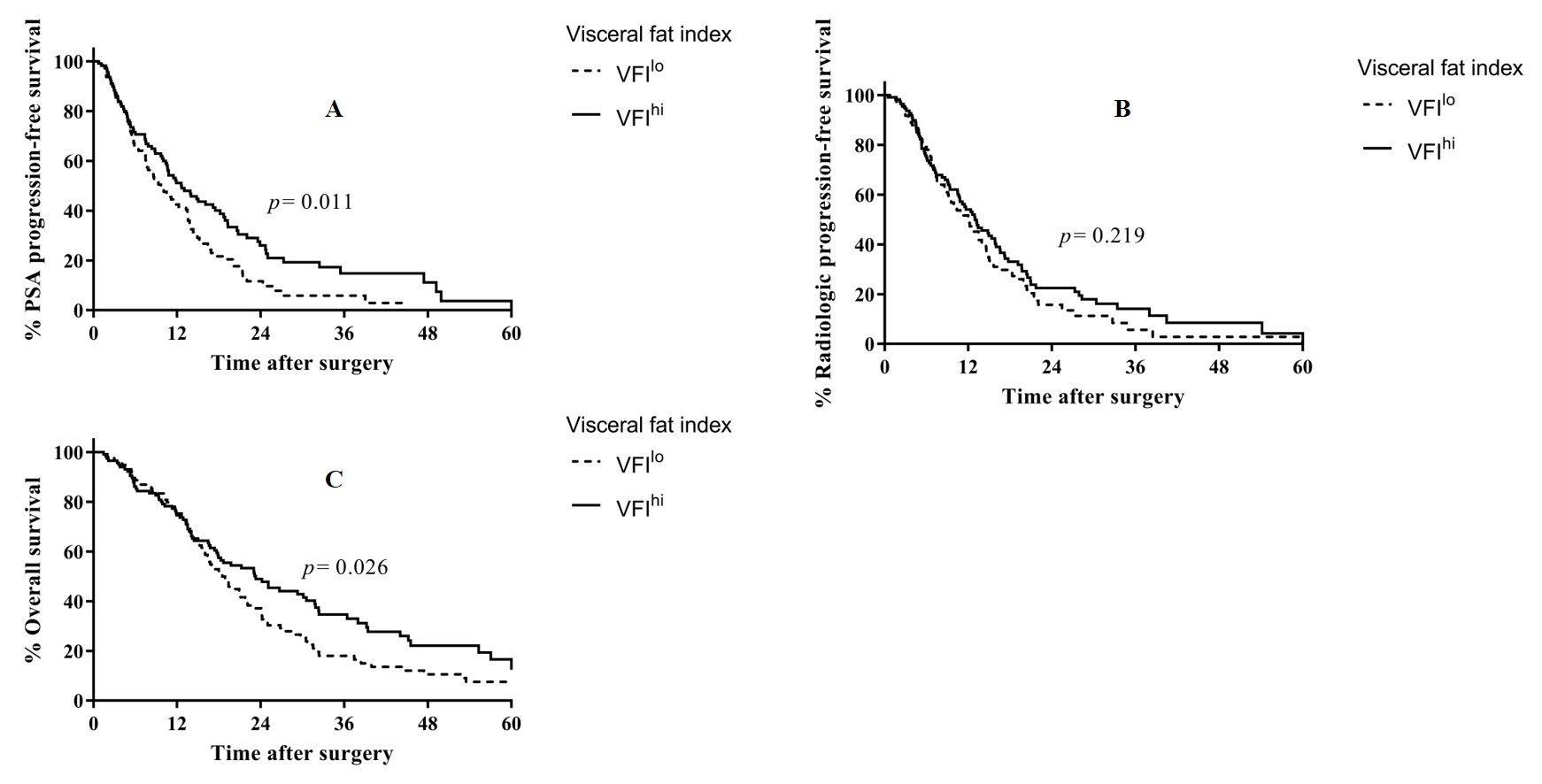

Supplement: Figure S1 — Outcomes based on visceral fat index in overall patients with castration-resistant prostate cancer from the time of diagnosis. (A) Prostate-specific antigen progression-free survival. (B) Radiologic progression-free survival. (C) Overall survival. [file Image_1.JPEG]

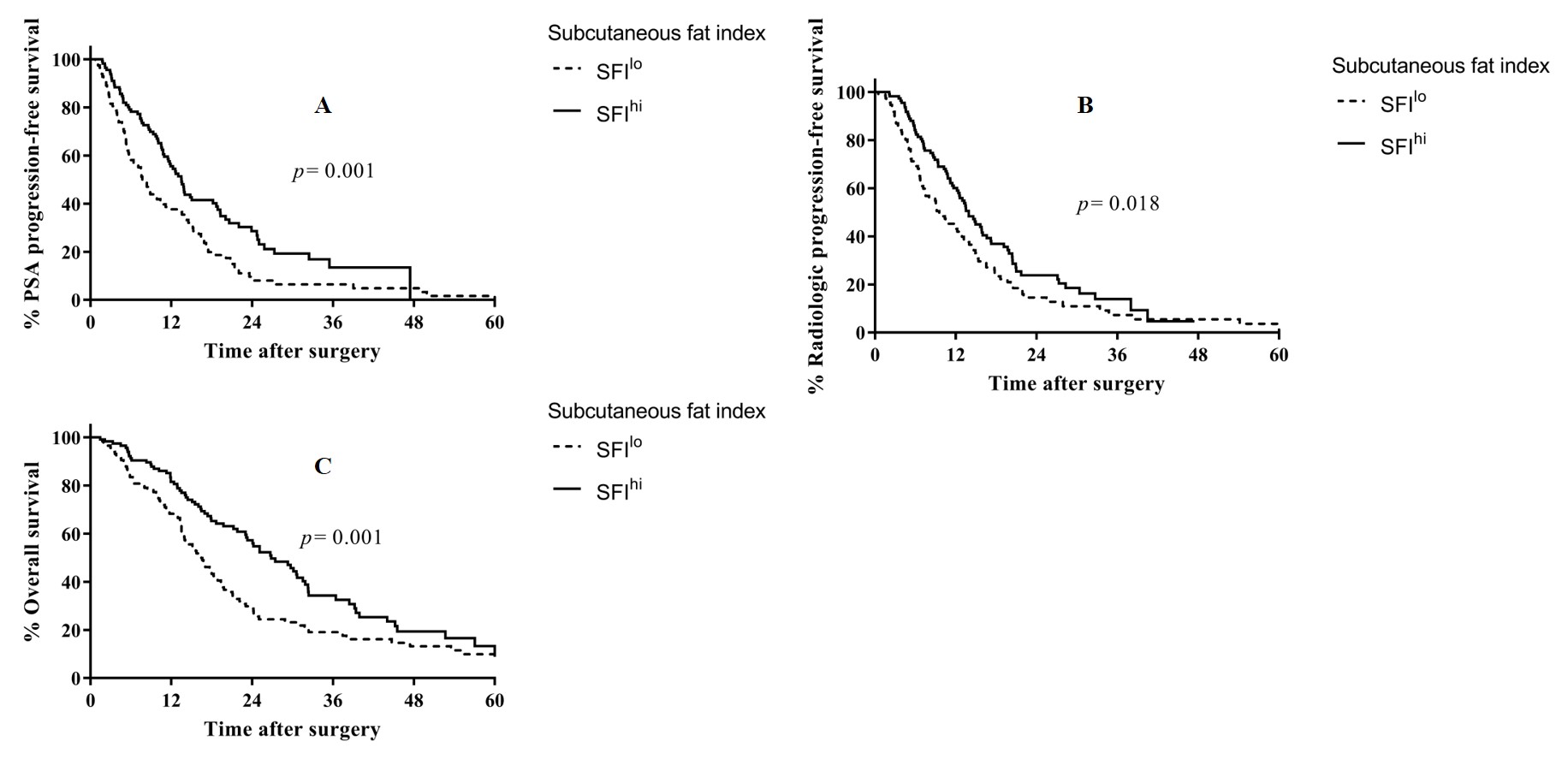

Supplement: Figure S2 — Outcomes based on subcutaneous fat index in overall patients with castration-resistant prostate cancer from the time of diagnosis. (A) Prostate-specific antigen progression-free survival. (B) Radiologic progression-free survival. (C) Overall survival. [file Image_2.JPEG]

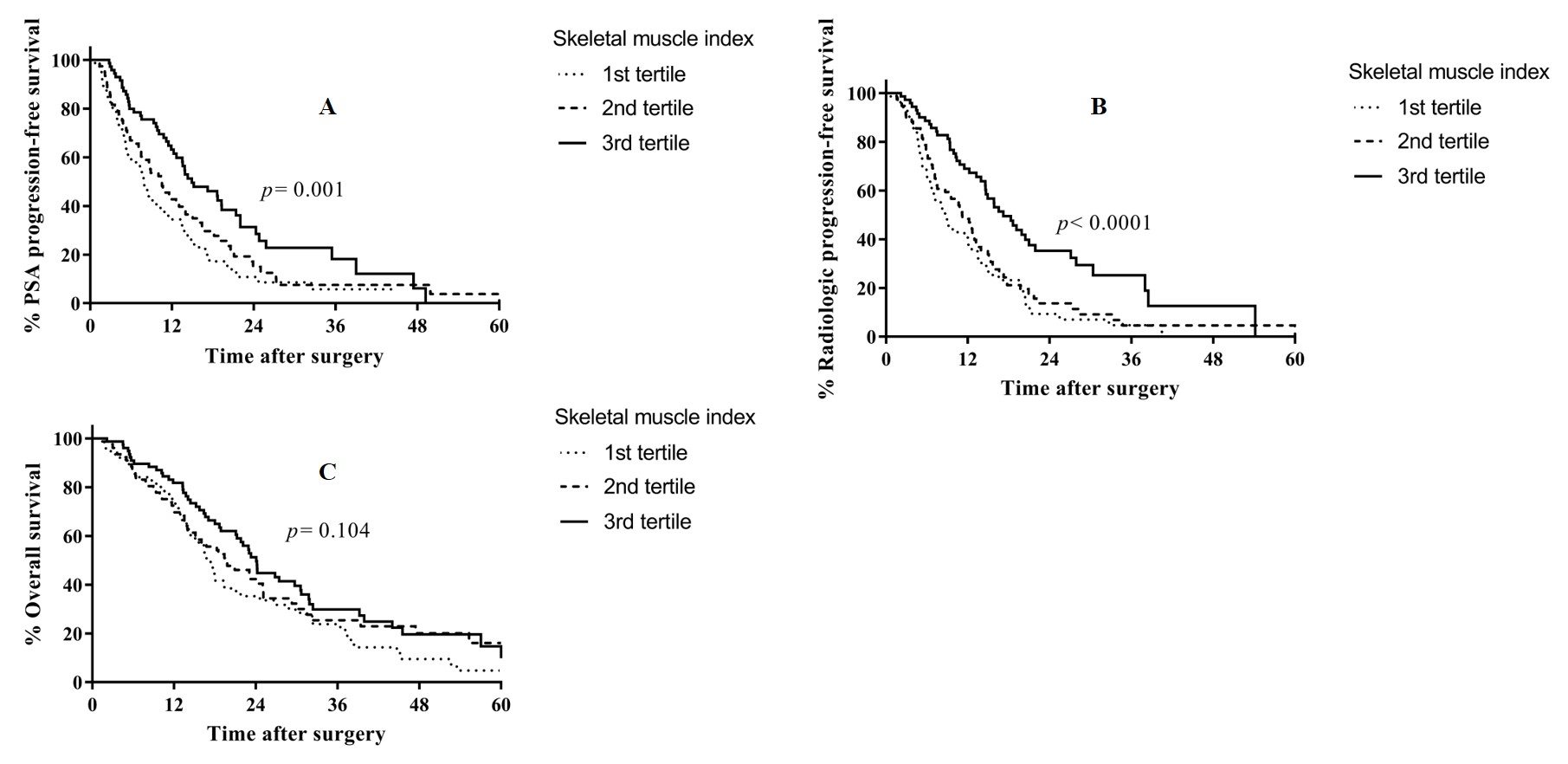

Supplement: Figure S3 — Outcomes based on skeletal muscle index tertiles in overall patients with castration-resistant prostate cancer from the time of diagnosis. (A) Prostate-specific antigen progression-free survival. (B) Radiologic progression-free survival. (C) Overall survival. 1st tertile: 26.73–46.92, 2nd tertile: 46.99–53.07, 3rd tertile: 53.09–77.24. [file Image_3.JPEG]

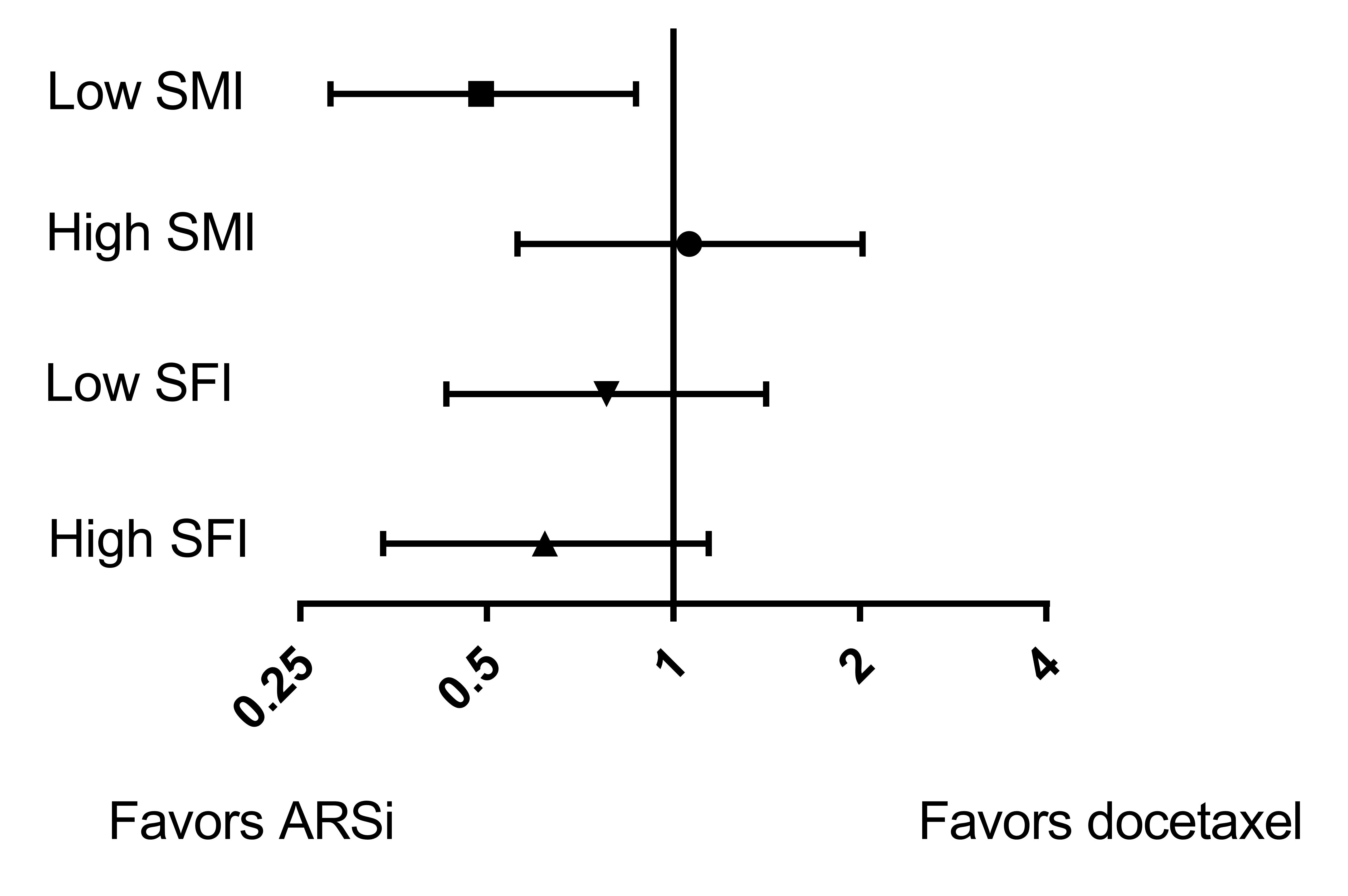

Supplement: Figure S4 — Treatment-specific hazards of radiologic progression. [file Image_4.JPEG]
